# Role of cardiac β_1_-adrenergic and A_1_-adenosine receptors in severe arrhythmias related to Parkinson's disease

**DOI:** 10.1016/j.clinsp.2023.100243

**Published:** 2023-07-15

**Authors:** Francisco Sandro Menezes-Rodrigues, Marcelo Pires de Oliveira, Erisvaldo Amarante Araújo, Henrique Ballalai Ferraz, Josef Finsterer, Efrain Olszewer, Murched Omar Taha, Carla Alessandra Scorza, Afonso Caricati-Neto, Fúlvio Alexandre Scorza

**Affiliations:** a Laboratory of Autonomic and Cardiovascular Pharmacology, Department of Pharmacology, Escola Paulista de Medicina (EPM), Universidade Federal de São Paulo (UNIFESP), São Paulo, SP, Brazil; b Neuroscience Discipline, Escola Paulista de Medicina (EPM), Universidade Federal de São Paulo (UNIFESP), São Paulo, SP, Brazil; c PostGraduate Program in Cardiology, Escola Paulista de Medicina (EPM), Universidade Federal de São Paulo (UNIFESP), São Paulo, SP, Brazil; d School of Medicine, Centro Universitário UNIFAS, União Metropolitana para a Educação e Cultura, Lauro de Freitas, BA, Brazil; e Klinikum Landstrasse, Messerli Institute, Vienna, Austria; f Fundação de Apoio à Pesquisa e Estudo na Área de Saúde (FAPES), São Paulo, SP, Brazil; g Department of Surgery, Escola Paulista de Medicina (EPM), Universidade Federal de São Paulo (UNIFESP), São Paulo, SP, Brazil

**Keywords:** Parkinson´s disease, Cardiac arrhythmias, Cardioprotection, β_1_-adrenoceptors, A_1_-adenosine receptors

## Abstract

**Aims:**

Although reduced life expectancy in Parkinson's Disease (PD) patients has been related to severe cardiac arrhythmias due to autonomic dysfunctions, its molecular mechanisms remain unclear. To investigate the role of cardiac β_1_-Adrenergic (β_1_AR) and A_1_-Adenosine (A_1_R) receptors in these dysfunctions, the pharmacological effects of stimulation of cardiac β_1_AR (isoproterenol, ISO), in the absence and presence of cardiac β_1_AR (atenolol, AT) or A_1_R (1,3-dipropyl-8-cyclopentyl xanthine, DPCPX) blockade, on the arrhythmias induced by Ischemia/Reperfusion (CIR) in an animal PD model were studied.

**Methods:**

PD was produced by dopaminergic lesions (confirmed by immunohistochemistry analysis) caused by the injection of 6-hydroxydopamine (6-OHDA, 6 μg) in rat striatum. CIR was produced by a surgical interruption for 10 min followed by reestablishment of blood circulation in the descendent left coronary artery. On the incidence of CIR-Induced Ventricular Arrhythmias (VA), Atrioventricular Block (AVB), and Lethality (LET), evaluated by Electrocardiogram (ECG) analysis, the effects of intravenous treatment with ISO, AT and DPCPX (before CIR) were studied.

**Results:**

VA, AVB and LET incidences were significantly higher in 6-OHDA (83%, 92%, 100%, respectively) than in control rats (58%, 67% and 67%, respectively). ISO treatment significantly reduced these incidences in 6-OHDA (33%, 33% and 42%, respectively) and control rats (25%, 25%, 33%, respectively), indicating that stimulation of cardiac β_1_AR induced cardioprotection. This response was prevented by pretreatment with AT and DPCPX, confirming the involvement of cardiac β_1_AR and A_1_R.

**Conclusion:**

Pharmacological modulation of cardiac β_1_AR and A_1_R could be a potential therapeutic strategy to reduce severe arrhythmias and increase life expectancy in PD patients.

## Highlights

• Pharmacological modulation of cardiac receptors can be an efficient therapeutic strategy to increase life expectancy in Parkinson's disease.

## Introduction

Clinically diagnosed by motor dysfunctions, such as bradykinesia, tremors at rest, and rigidity, Parkinson's Disease (PD) is the second most common neurodegenerative disease worldwide [1]. PD is pathophysiologically characterized by degeneration and death of dopaminergic neurons in the *Substantia* Nigra pars compacta (*SNc*) associated with the development of intraneuronal aggregates of α-synuclein protein (Lewy body) [ 1,2]. As PD incidence is higher in older individuals, the aging of the world population can considerably increase the number of PD cases in the coming years [3]. A recent report by the United Nations-World Population Ageing (2019) indicates that the number of individuals over 65 years old worldwide may double from 703 million in 2019 to about 1.5 billion in 2050, in addition about 426 million of these people are expected to be over 85 years old [4]. In this worrisome scenario, there is no proven disease-modifying therapy to avoid PD progression and severity.

In addition to motor dysfunctions, several non-motor dysfunctions are associated with PD, such as autonomic and cardiovascular dysfunctions [5]. These dysfunctions are importantly involved in PD pathophysiology, causing cardiac arrhythmias and sudden death [5]. These dysfunctions have been identified at all stages of PD and diagnosed in about 60% of PD patients, constituting a leading cause of death due to PD [5], [6], [7], [8], [9], [10]. Several studies have shown that mortality in PD patients is significantly higher than that seen in the general population [11], [12], [13]. A recent review by the research group showed that from a total of about 97,000 scientific articles on PD, 1,650 were related to mortality in PD [5]. Despite a robust body of clinical evidence suggesting a high incidence of cardiovascular disorders associated with reduced life expectancy in PD patients [5,6,9,10,[17], [18], [19], [20], [21], [22], [23]], its pathophysiology remains unclear.

Many important advances in PD pathophysiology were observed in the last decades, mostly derived from studies using PD animal models that mimic both motor and non-motor symptoms observed in humans [14], [15], [16]. The animal model generated by the administration of the neurotoxin 6-Hydroxydopamine (6-OHDA) directly into the Striatum (ST) of rats is widely used and beyond motor deficits of PD, also reproduces a broad spectrum of non-motor comorbidities, decisively contributing to expanding the knowledge of the PD pathophysiology, especially in relation to molecular mechanisms involved in autonomic and cardiovascular dysfunctions involved in this neurodegenerative disease [14], [15], [16].

It is well established that autonomic regulation of cardiac function is mainly mediated by β_1_-Adrenergic Receptors (β_1_AR) located on the plasma membranes of cardiac cells, which constitutes the major βAR subtype (75% to 80%) expressed in the mammalian heart [24], [25], [26]. When stimulated by the neurotransmitter Noradrenaline (NA) released by intracardiac sympathetic neurons, cardiac β_1_AR promotes activation of Adenylyl Cyclase (AC) that, in turn, increases intracellular adenosine 3`,5`-Cyclic Monophosphate (cAMP) levels and consequent activation of cAMP-dependent Protein Kinases (PKA) [24], [25], [26]. In its activated state, PKA phosphorylates several cell proteins, including troponin I, L-type voltage-gated Ca^2+^ channel (L-type Cav) and phospholamban, increasing the cardiac chronotropic and inotropic responses [24], [25], [26].

Cardiac β_1_AR-mediated chronotropic responses are finely modulated by Adenosine Triphosphate (ATP) released with NA from intracardiac sympathetic neurons [26], [27], [28], [29]. ATP released is enzymatically converted to Adenosine (ADO), which activates cardiac A_1_ Adenosine Receptors (A_1_R), leading to attenuation of positive chronotropic responses stimulated by β_1_AR [26], [27], [28], [29]. In addition, cAMP released to the extracellular medium from cardiac cells during sympathetic stimulation leads to an increase in extracellular levels of ADO, increasing the A_1_R activation [26], [27], [28], [29]. Several studies suggest that the cellular action of cAMP and PKA are essential for cardioprotective responses mediated by cardiac β_1_AR and A_1_R [26], [27], [28], [29], [30], [31], [32]. Thus, have been proposed that this adrenergic-purinergic communication responsible by fine regulation of cardiac function could importantly contribute to cardioprotective responses in different pathophysiological conditions [26], [27], [28], [29], [30], [31], [32].

Although β_1_AR and A_1_R have an important physiological role in the regulation of cardiac function [26], [27], [28], [29], their role in cardiac autonomic dysfunctions associated with PD is unknown. To investigate the pathophysiological role of β_1_AR and A_1_R in cardiac autonomic dysfunctions associated with PD, we studied the effects of βAR agonists (isoproterenol, ISO), and selective antagonists of β_1_AR-selective (atenolol, AT) or A_1_R (1,3-Dipropyl-8-Cyclopentyl Xanthine, DPCPX), on the incidence of Ventricular Arrhythmias (VA), Atrioventricular Block (AVB) and Lethality (LET) induced by Cardiac Ischemia/Reperfusion (CIR) in the 6-OHDA model of PD.

## Materials and methods

### Animals

Adult (16‒20-week-old) male Wistar rats were provided by the Center for Development of Experimental Models in Medicine and Biology (CEDEME) of Federal University of São Paulo (UNIFESP). Rats were maintained under standard conditions of nutrition, hydration, temperature, humidity, and luminosity until the moment of experimentation. All experimental procedures were approved by the Ethical Committee on Animal Use of UNIFESP (CEUA nº 2367271115) and were in accordance with the regulations of the National Council for the Control of Animal Experimentation (CONCEA, Brazil).

### Induction of Parkinson's disease (PD) model

In this study, we have chosen the PD model induced by 6-OHDA to reproduce in laboratory conditions the initial stages of PD development in humans, as previously proposed by several studies [14], [15], [16]. Due to its poor ability to cross the blood-brain barrier, 6-OHDA is injected into the ST to induce degeneration and death of dopaminergic neurons [14], [15], [16]. Inside the ST, 6-OHDA is uptaken by dopaminergic neurons through the Dopamine Neuronal Transporter (DAT), causing degeneration and death in these neurons due to increased formation of H_2_O_2_ and free radicals, and inhibition of complex I and IV activity in the mitochondrial respiratory chain [14], [15], [16]. To produce a PD model in rats, the surgical protocol as described by Real et al. was used [33]. As such, animals’ skulls were shaved and their skin was cleaned with 70% alcohol. Then, they were anesthetized with ketamine (150 mg/kg, i.p.) and xylazine (10 mg/kg, i.p.), and positioned in the stereotaxic apparatus by their ear canals. A longitudinal midline incision was done, the subcutaneous and muscle tissues were separated, and bregma and lambda landmarks in the rat skull were exposed [33]. Striatal injections into a single cerebral hemisphere were performed in two-point coordinates from the bregma were defined: 1^st^ point: AP Bregma, ML − 2.7 mm, and DV − 4.5 mm; 2^nd^ point: AP + 0.5 mm, mL − 3.2 mm, and DV − 4.5 mm [24]. Then, a thin hole was opened in the skull over the target area, using a hand-held drill, and, with a Hamilton micro-syringe (5 μL), 0.5 μL of a solution containing 3 μg 6-OHDA in 0.3% ascorbic acid (6 μg/μL) was injected into each point (6-OHDA group) [33]. In order to assure solution diffusion, the syringe was kept in place for 5 min. Then, the incision was sutured, and the rats were returned to their cages for recovery. One group of rats (control group) was subjected to all these procedures, except that 0.9% Saline Solution (SS) was injected instead of 6-OHDA. After seven days of 6-OHDA or SS injection, rats were submitted to CIR protocol and Tyrosine Hydroxylase (TH) immunoreactivity to evaluate cell death in ST and *SNc*.

### Evaluation of cardiac arrhythmias induced by CIR

Several studies showed that reduced cardiac sympathetic activity increases the incidence of fatal cardiac arrhythmias in PD [9,20,21]. The CIR protocol has been useful to study cardiac dysfunctions related to different pathological conditions and cardioprotective strategies [26,[30], [31], [32]]. To investigate the pathophysiological role of β_1_AR and A_1_R in cardiac autonomic dysfunctions associated with PD, we studied the effects of ISO (non-selective βAR agonist), in the absence and presence of AT (β_1_A-selective antagonist) and DPCPX (A_1_R-selective antagonist), on the incidence of CIR-induced cardiac arrhythmias (VA and AVB) and LET in the 6-OHDA model of PD.

The CIR protocol used to induce cardiac arrhythmias was based in the methodology described by Tavares et al [34]. Thus, rats were anesthetized with urethane (1.25 g/kg, i.p.) and mechanically ventilated (Harvard Apparatus, Boston) [34]. After the stabilization period (15 min), the heart was gently exposed by left thoracotomy to mechanical occlusion of the left anterior descending artery using a plastic rod (ischemia). After ischemia (10 min), the rod was removed to allow coronary reperfusion for 75 min. The incidence of VA and AVB induced by CIR was evaluated by Electrocardiogram (ECG) analysis performed before and during CIR protocol in 6-OHDA and control rats, [34]. with the AqDados 7.02 software, while raw data were analyzed with the AqDAnalysis 7 software (Lynx Tecnologia Ltda, Brazil) [25]. Using this computational analysis, the incidence of VA, AVB and LET induced by CIR was determined before and after treatment with 0.5 mg/kg, i.v. ISO 5 min before ischemia in 6-OHDA (6-OHDA + ISO group) and control (C + ISO group) animals. To evaluate the role of cardiac β_1_-AR and A_1_-R in cardiac arrhythmias induced by CIR in the 6-OHDA model of PD, ISO effects were studied in the absence or presence of blockade of β_1_-AR with AT (10 mg/kg) or A_1_R with DPCPX (100 μg/kg). These selective antagonists were intravenously administrated 5 min before ISO in Control (C) and 6-OHDA rats. The rats were divided in eight experimental groups: C, C+ISO, C+AT+ISO, C+DPCPX+ISO, 6-OHDA, 6-OHDA+ISO, 6-OHDA+AT+ISO and 6-OHDA+DPCPX+ISO.

### Immunostaining

Physiologically, DA synthesis is limited by Tyrosine Hydroxylase (TH) activity. This enzyme converts tyrosine actively transported to the cytosol of sympathetic neurons into Dihydroxyphenylalanine (DOPA), whereas aromatic Amino Acid Decarboxylase (AADC) converts DOPA into DA [14], [15], [16]. Catecholaminergic neurons are effectively labeled by TH which is an excellent biological marker of nigrostriatal dopaminergic neurons [14], [15], [16]. 6-OHDA and control rats were anesthetized with urethane (1.25 g/kg, i.p.) and decapitated for brain removal. Brains were fixed in 4% paraformaldehyde and placed in a 30% sucrose solution. Free-floating 30 μm coronal sections were treated with 1% H_2_O_2_ in PBS (0.1 M sodium phosphate buffer, Ph 7.2) for 30 min. After washing, sections were incubated overnight with primary antibody (anti-TH; 1:1000; MAB5280, Chemicon, USA) in PBS containing 0.3% Triton X-100 and 5% normal donkey serum. Next, sections were incubated with secondary antibody in PBS with 0.3% Triton X-100 for 120 min (1:200; Jackson Labs, West Grove, PA, USA), and then treated with avidin-biotin-peroxidase complex (ABC Elite; Vector Labs, Burlingame, CA, USA). The reaction was revealed with 0.05% 3-3-diaminobenzidine tetrahydrochloride containing 0.01% H_2_O_2_ in PBS, and sections were mounted on gelatin-coated slides. Five digital images covering the entire extension of ST and *SNc* were acquired using a Nikon E1000 microscope and a Nikon DMX1200 digital camera. Integrated optical density using Image J software (NIH, Bethesda, MD, USA) was evaluated. The percentage of cell survival was determined by the ratio of the total number of cells on the right (experimental) and left (contra-lateral) ST from 6-OHDA and control animals.

### Drugs and reagents

The neurotoxin 6-hydroxydopamine (6-OHDA) was purchased from Sigma-Aldrich (USA). Ketamine, xylazine, and urethane were purchased from Syntec (Brazil). Other drugs were purchased from Sigma-Aldrich (USA).

### Statistical analysis

For statistical analysis, GraphPad Prism 6.0 software (GraphPad Software Inc., CA, USA) was used. The incidence of VA, AVB, and LET in 6-OHDA and control rats were compared with Fisher's exact test [34]. Mean values of the integrated optical density for TH immunostaining in 6-OHDA and control rats were compared with non-paired Student's *t*-test with Welch's correction. Results were expressed as mean ± Standard Error Mean (SEM). Values of p < 0.05 were considered statistically significant.

## Results

### TH Immunoreactivity

**Fig. 1:**
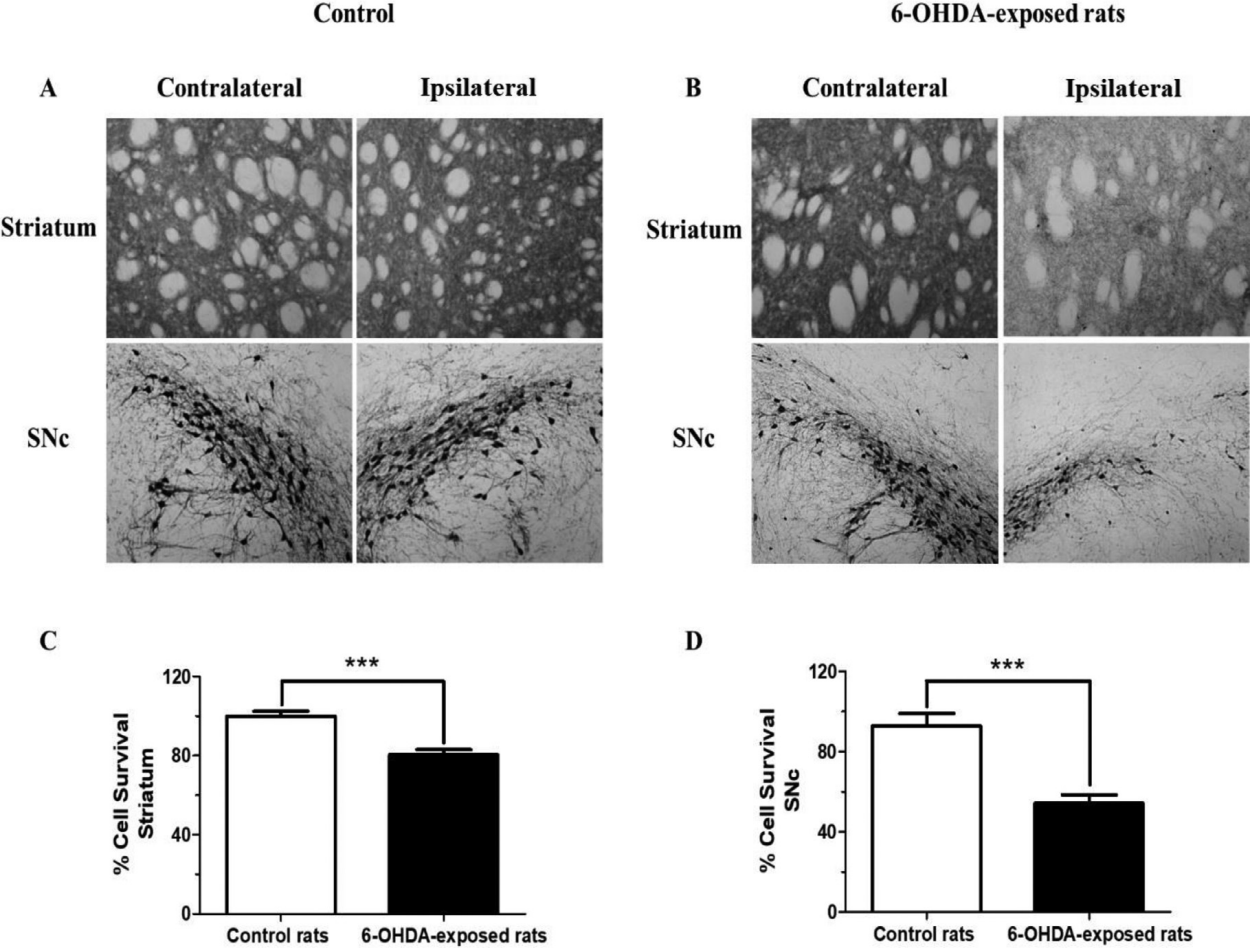
Representative images showing TH-immunostained sections of ST and SNc from 6-OHDA and control rats. (A) TH-immunoreactivity in the ST (top) and SNc (bottom) of the contralateral (left) and ipsilateral (right) sides shows no reduction in TH-positive cells in the control rats. (B) Seven days after 6-OHDA, TH-immunoreactivity in the ST (top) and SNc (bottom) of the ipsilateral (right) side shows significant decrease in TH-immunostaining compared to the contralateral (left) side in rats that were unilaterally injected with 6-OHDA. Quantification of TH-immunostaining in the experimental side of the ST (C) and SNc (D) shows a significant decrease in the 6-OHDA-exposed rats compared to control rats. ***Statistically different from the control (p < 0.001).

TH immunoreactivity in ST and *SNc* from 6-OHDA and control rats revealed distinct (Fig. 1 A and B). The percentage of dopaminergic neurons in the 6-OHDA-lesioned ST (80.50 ± 2.51%) was significantly lower (∼20%) compared to control (99.99 ± 2.50%) rats (Fig. 1C). The percentage of dopaminergic neurons in the *SNc* from 6-OHDA rats (54.25 ± 4.22%) was also significantly lower (∼45%) compared to control (92.85 ± 6.29%) (Fig. 1D). In accordance with several studies [14], [15], [16], this reduction in dopaminergic neurons confirmed the nigrostriatal lesion in 6-OHDA rats.

### Incidence of AV, AVB and LET induced by CIR in 6-OHDA rats

**Fig. 2:**
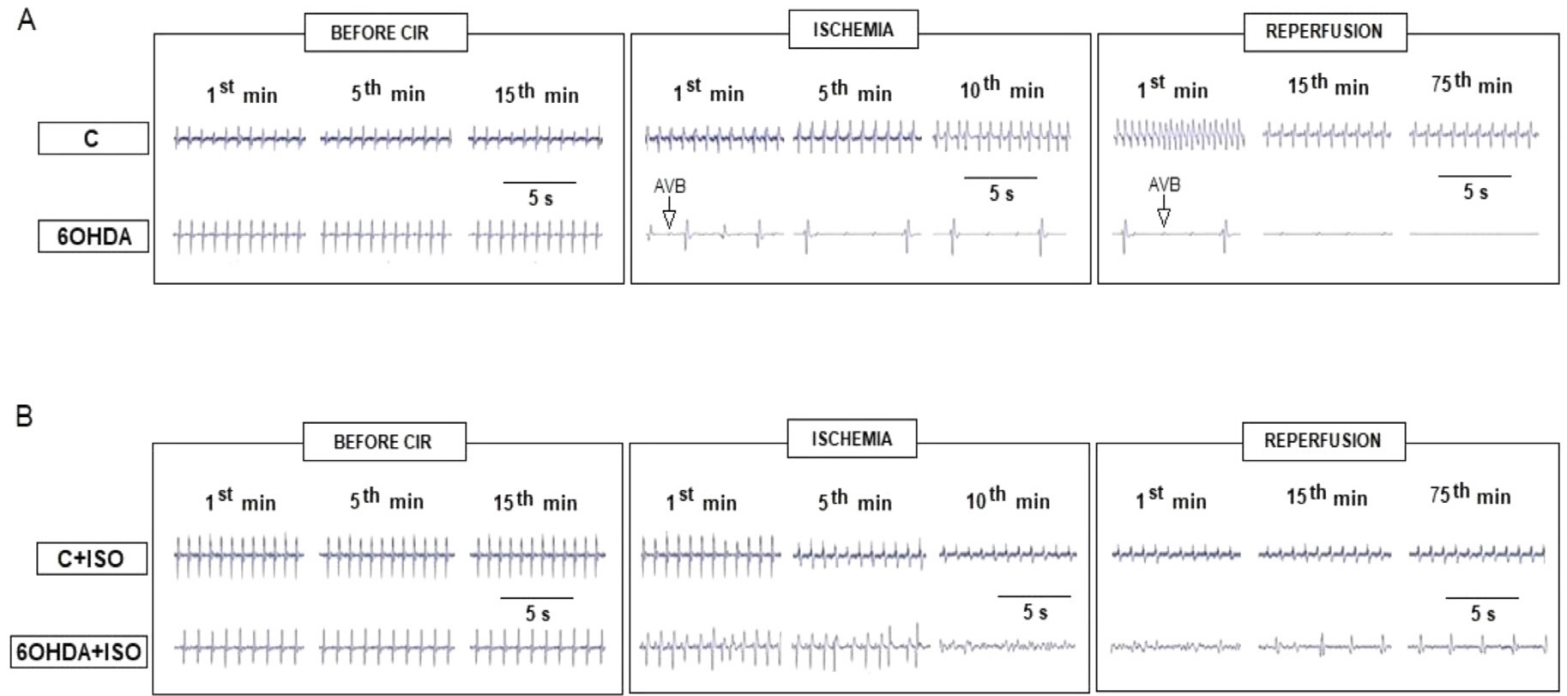
Typical ECG records showing the VA and AVB induced by CIR in 6-OHDA and control rats. The baseline values of heart rate recorded before CIR (in rest) showed no significant differences between 6-OHDA and control (C) groups. (A) With less than 1 min after cardiac ischemia, arrhythmias were observed in 6-OHDA, but not in C rats. Arrhythmias tended to worsen over time in the 6-OHDA group, which evolved to AVB (see ECG obtained in 1^st^, 5^th^ and 10^th^ min ischemia, and 1^st^ and 75^th^ min reperfusion) in 92% (11/12) of these rats. In contrast, the incidence of AVB was lower in C rats. (B) Incidence of VA, AVB and LET was significantly reduced by treatment with ISO (0.5 mg/kg, i.v., before ischemia) in 6-OHDA + ISO and C + ISO groups.

**Fig. 3:**
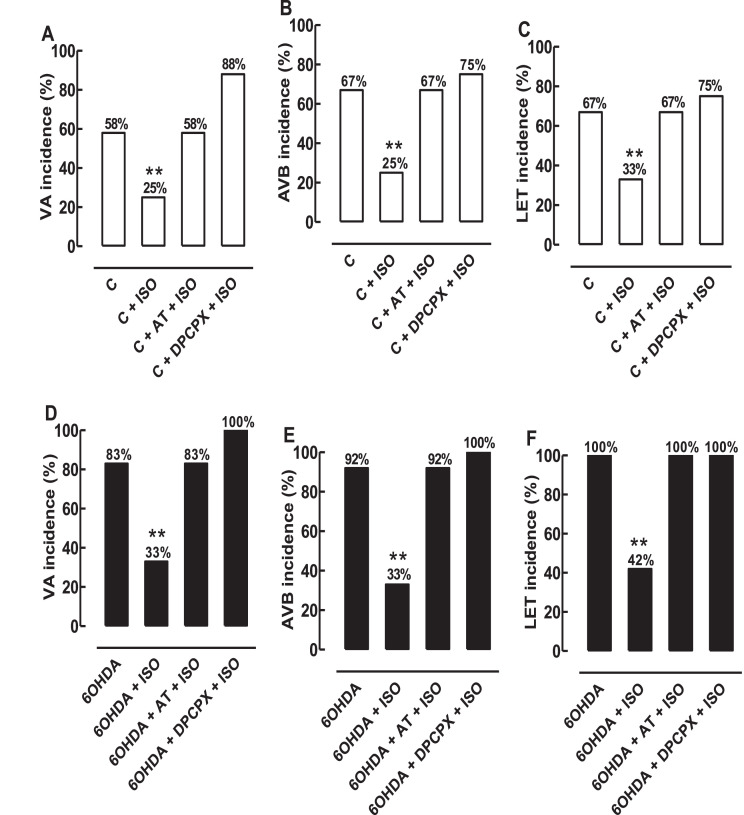
Histograms representing the incidence of VA, AVB, and LET induced by CIR, before and after the treatment with ISO and AT, in 6-OHDA and control rats. The incidence of VA (A, D), AVB (B, E) and LET (C, F) was significantly higher in 6-OHDA than in control group. The treatment with ISO immediately before ischemia significantly decreased the incidence of VA, AVB and LET in the 6-OHDA + ISO and C + ISO group. Pretreatment both AT (10 mg/kg, i.v., before ischemia) and DPCPX (100 µg/kg, i.v., before ischemia) abolished these ISO effects in all groups, confirming the involvement of both β_1_AR and A_1_R in this cardioprotective response. Values were expressed as mean ± SEM obtained in 6-OHDA (n = 12) and control (n = 12) rats. **Statistically different from the 6-OHDA or control rats (p < 0.01).

Although heart rate did not differ between 6-OHDA (335 ± 13 bpm, n = 12) and control (346 ± 15 bpm, n = 12) rats before the CIR protocol, this parameter varied considerably during CIR induction. Typical ECG records (Fig. 2) of 6-OHDA and control rats show that, after 1 min of ischemia, the VA evolved to AVB (6/12; 50%) in 6-OHDA rats, while no VA and AVB were detected in control rats. After 5 min of ischemia, an increase in the incidence of AVB was detected in 6-OHDA rats (8/12; 67%) resulting in death in 33% (4/12) of them, while there was no AVB (Fig. 2) or death among control rats. After 10 min of ischemia, the incidence of AVB significantly increased in the 6-OHDA group (11/12; 92%), while there was VA but no AVB (Fig. 2) or death in control rats. After 1 min of reperfusion, the incidence of AVB remained high in 6-OHDA rats (11/12; 92%), resulting in the death of the remaining 67% (8/12) of 6-OHDA rats before the 15^th^ min of reperfusion. In control rats, the increase in VA incidence (7/12; 58%) during reperfusion resulted in higher of AVB incidence (8/12; 67%). In consequence, the incidence of LET in these rats was 67% (8/12) at the end of 75 min of reperfusion. The incidence of VA, AVB and LET was significantly higher in 6-OHDA (83%, 92% and 100%, respectively) than in control rats (58%, 67% and 67%, respectively), suggesting that this PD model was more susceptible to severe and fatal arrhythmias induced by CIR (Fig. 3).

Although the incidence of VA, AVB, and LET were elevated at the end of the CIR protocol, treatment with 0.5 mg/kg i.v. ISO administered 5 min before ischemia significantly reduced this incidence in 6-OHDA and control rats. The incidence of VA was reduced from 83% (10/12) in the 6-OHDA group to 33% (4/12) in the 6-OHDA + ISO group (p < 0.01), and from 58% (7/12) in the control group to 25% (3/12) in the C + ISO group (p < 0.01). The incidence of AVB was significantly reduced from 92% (11/12) in the 6-OHDA group to 33% (4/12) in the 6-OHDA + ISO group (p < 0.01), and from 67% (8/12) in the control group to 25% (3/12) in the C + ISO group (p < 0.01). Due to this cardioprotective effect mediated by ISO, the incidence of LET significantly decreased from 100% (12/12) in the 6-OHDA group to 42% (5/12) in the 6-OHDA + ISO group (p < 0.01), and from 67% (8/12) in the control group to 33% (4/12) in the C + ISO group (p < 0.01). These ISO effects were abolished by blockade of pretreatment with 10 mg/kg i.v. AT 5 min before ischemia in the 6-OHDA + AT + ISO and C + AT + ISO groups (Fig. 3), confirming the involvement of β_1_AR in cardioprotective responses induced by ISO.

In addition, the ISO effects also were abolished by pretreatment with 100 µg/kg i.v. DPCPX 5 min before ischemia in the 6-OHDA + DPCPX + ISO and C + DPCPX + ISO groups (Fig. 3), confirming the involvement of A_1_R in cardioprotective responses induced by ISO.

## Discussion

Although autonomic dysfunctions that cause severe and fatal cardiac arrhythmias have been associated with reduced life expectancy in patients with PD [9,17,[20], [21], [22], [23]], its pathophysiology remains unclear, thus making pharmacotherapy more difficult. In this work, we showed that treatment with ISO significantly reduced the incidence of AVB and LET induced by a CIR in an animal model of PD produced by nigrostriatal lesions induced by 6-OHDA, indicating that moderate sympathomimetic pharmacological stimulation of cardiac β_1_AR could reduce the incidence of fatal arrhythmias in PD. The present findings contribute to the understanding of the participation of the β_1_AR and A_1_R in the cardiac dysfunction of Parkinsonian rats and to the future development of new therapeutic strategies to reduce the incidence and severity of cardiac disorders.

The heart rate variability and delayed orthostatic hypotension resulting from cardiac autonomic dysfunctions constitute an important risk factor for PD in humans [6,9,35,36]. Initially attributed to damage in distinct components of the central and peripheral nervous systems, especially in cardiac sympathetic neurons [17,[20], [21], [22], [23], 36], these dysfunctions are variable and affect both sympathetic and parasympathetic regulation of cardiovascular activity in PD [20,37,38]. Increased incidence of severe and fatal arrhythmias in PD patients has been related to reduced sympathetic and increased parasympathetic activity [9,17,[20], [21], [22], [23]]. However, the pathophysiology and possible pharmacotherapy of these autonomic dysfunctions remain unclear.

A recent populational-based study by Hong et al [39]. identified Atrial Fibrillation (AF) as a significant comorbidity in the preclinical stage of PD in human patients, which led authors to suggest AF as a potential premotor predictive biomarker since the risk of AF was significantly lower in later stages of PD [39]. Autonomic nervous system abnormalities are frequent features already observed in prodromal PD causing a plethora of non-motor symptoms. Growing evidence shows that PD individuals exhibit a robust decrease in HRV parameters in comparison to healthy controls, even before PD diagnosis with the onset of motor deficits [40]. Our group and others described similar HRV changes in animal models of PD, reproducing human conditions [41,42]. In the present study, cardiac function was assessed in parkinsonian rats the relationship between sympathetic dysfunction triggers a decrease in purinergic activity since sympathetic dysfunction culminates in a decrease in cAMP production in the intracellular environment, and this reduction in cAMP production leads to a lower efflux of this second messenger to the extracellular environment, causing a decrease in the conversion of cAMP into adenosine and, therefore, decreasing the adenosine autocrine activity on A_1_R cardioprotective receptors, these events can be observed even at an early stage (early stage, mimicking preclinical stage of human patients) of the disease.

Intra and extracardiac sympathetic denervation, as well as attenuation of arterial baroreflexes, appear to be causal factors for these cardiovascular autonomic dysfunctions in PD patients [6,43]. Loss of baroreceptor sensitivity in PD patients has been documented by spectral analysis of heart rate (R-R interval) and systolic arterial pressure variability [43]. A sustained drop in systolic pressure of at least 20 mm.Hg and/or a sustained diastolic drop of at least 10 mm.Hg has been observed in PD patients within the first 3 min after standing up [6]. In addition to the loss of baroreflexes, intra and extracardiac sympathetic denervation may be directly involved in the high incidence of severe cardiac arrhythmias in PD patients [9], as observed in decreased cardiac sympathetic activity in the PD model induced by 6-OHDA [20]. Although the molecular mechanisms involved in sympathetic dysfunctions in PD remain unknown, it is possible that cardiac autonomic receptors have an important role in these dysfunctions.

Cardiac β_1_AR and A_1_R have an important physiological role in the neurogenic regulation of cardiac function [24], [25], [26], [27], [28], [29]. In addition, β_1_AR and A_1_R are involved in cardioprotective responses as well [26,[30], [31], [32]]. The role of cardiac β_1_AR and A_1_R in cardiac autonomic dysfunctions in PD is unknown, however, it is possible that the function of these receptors is altered in PD. Then, pharmacological modulation of cardiac β_1_AR and A_1_R could be useful therapeutic strategies to decrease the incidence of severe and fatal arrhythmias in patients with PD [5,6]. 6-OHDA rats were more susceptible to severe and fatal arrhythmias when subjected to a CIR protocol compared to control animals, due to deregulation of autonomic control of cardiac activity aggravated by CIR [9,17,[20], [21], [22], [23], 36]. Stimulation of cardiac β_1_AR in an animal model of PD reduced the incidence the severe and fatal arrhythmias induced by CIR [26,44].

Nitric Oxide (NO), PKA, and PI_3_K are essential for cardioprotective responses mediated by cardiac β_1_AR [26,[30], [31], [32], [44], [45], [46], [47], [48], [49]]. Stimulation of cardiac β_1_AR increases NO biosynthesis through activation of the GC/cGMP/PKG pathway, which increases the activity of mK_ATP_ channels, preserving mitochondrial bioenergetics, attenuating cardiac excitation-contraction decoupling, and thus reducing the incidence of severe arrhythmias and death of cardiac cells [30], [31], [32]. Several drugs can increase intracellular cyclic AMP, among which β_1_AR agonists such as ISO stand out. In cardiac cells, ISO activates Gs/AC/cAMP/PKA signaling, that phosphorylates L-type Cav, resulting in increased Ca^2+^ influx with positive chronotropic and inotropic effects [49,50]. After the increase in intracellular cAMP, the efflux of cAMP to the extracellular medium occurs through the Multi-drug Resistant Protein 4 channel (MRP4) [51,52]. This is followed by the extracellular conversion of cAMP to ADO by the serial actions of Ecto-Phosphodiesterase (EPDE) and Ecto-5′-Nucleotidase (ENT) [52]. This mechanism of extracellular ADO biosynthesis may provide hormonal control of ADO levels in the cell-surface biophase in which ADO receptors reside. Simultaneous addition of an inhibitor of the membrane transporter MRP4 and ISO was able to increase the intracellular concentration of cAMP when compared with the addition of ISO alone [51,52].

**Fig. 4:**
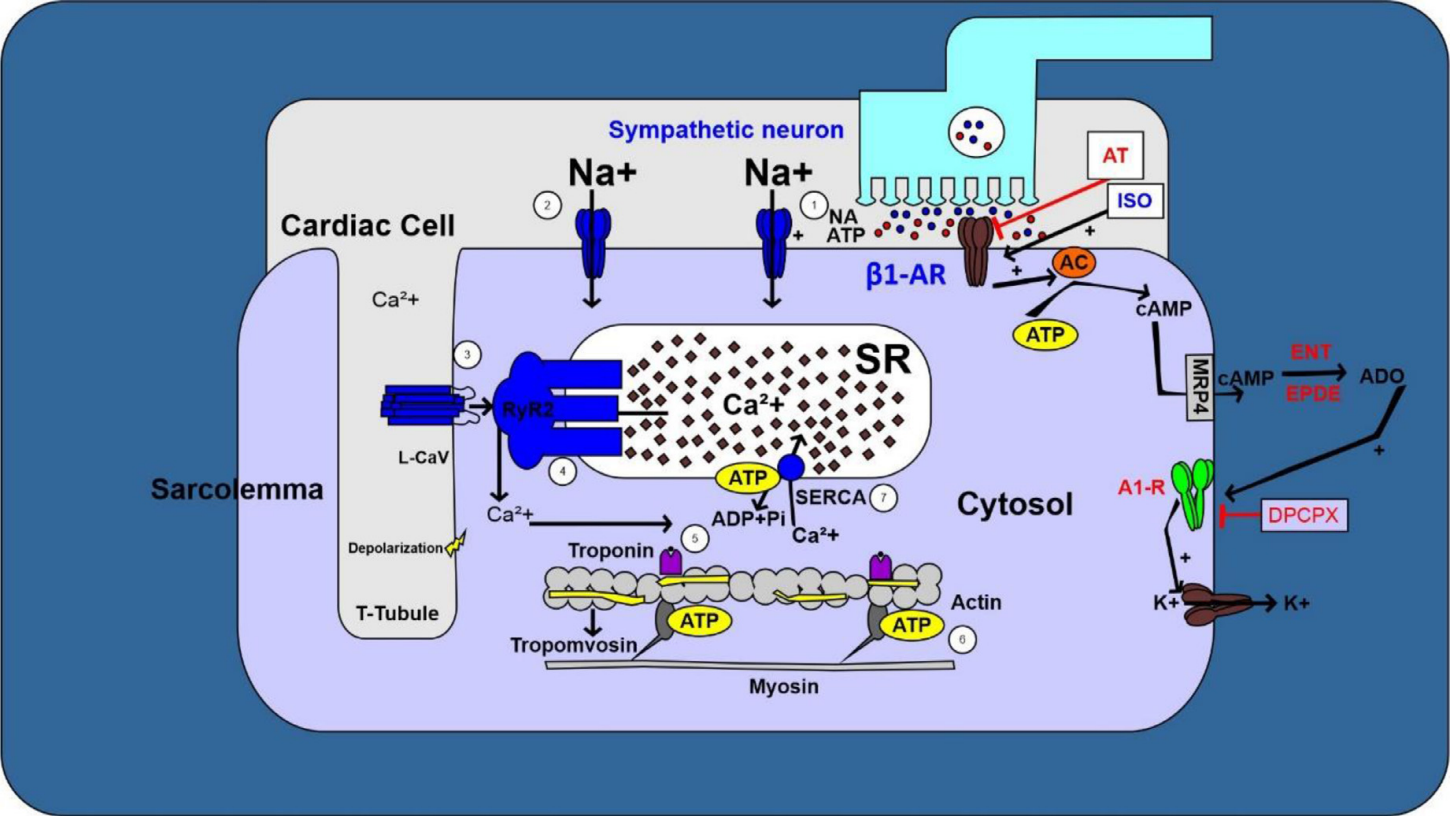
Molecular mechanisms involved in the sympathetic regulation of cardiac function mediated by β_1_AR and A_1_R. The β_1_AR are the major subtype expressed in the cardiac cells. When stimulated, these receptors coupled to Gs promote an increase in intracellular levels of cAMP through the activation of membrane enzyme AC. cAMP activates PKA that phosphorylates L-type Cav (L-Cav), resulting in increased Ca^2+^ influx with positive chronotropic and inotropic response. The increase in intracellular cAMP stimulates cAMP transport to the extracellular due to action of MRP4. In extracellular, cAMP is conversed to ADO due to enzymatic action of EPDE and ENT. This purinergic signaling involved action of ADO in A_1_Rfinely modulates functional response of β_1_AR in cardiac cells.

Thus, the communication between the adrenergic and purinergic pathways, which act together to regulate cardiac chronotropism, since the adrenergic pathway increases heart rate, while the A_1_R-mediated purinergic pathway attenuates sympathetic activity in the heart and, therefore, may offer a cardioprotective effect, especially when adrenergic activity is increased. In this context, activation of A_1_R, which is coupled to Gi, is a negative feedback mechanism finely controls intracellular cAMP levels and modulates cardiac chronotropism and dromopism [29], as well as indirect anti-β_1_AR actions [29,[53], [54], [55]] The molecular mechanisms involving sympathetic regulation of cardiac function mediated by β_1_AR and A_1_R were illustrated in Figure 4.

A_1_R was found in rodent myocardium and AC was found to be coupled to these receptors in ventricular membranes [[27], [28], [29], 31,[56], [57], [58], [59], [60]]. The existence and action of the cAMP-ADO extracellular pathway could explain the inhibitory effect promoted by ADO after ISO-induced AC activation in cardiac membranes [57], as well as elucidate the reason why hearts perfused with catecholamines prevention occurs with respect to total mechanical responsiveness [58]. Under stress conditions, such as hypoxia or ischemia, the increase in extracellular ADO levels is responsible for cardioprotective effects, which, at least in part, involve the activation of Gi-coupled A_1_R [59,60]. Pharmacological stimulation of cardiac β_1_AR results in indirect stimulation of cardiac A_1_R, through the conversion of cAMP into ADO in the extracellular medium, which in turn promotes activation of these A_1_R. These are responsible, in part, for the cardioprotective mechanisms that so reduce the incidence of severe CIR-induced cardiac arrhythmias responsible for death in PD model animals.

## Conclusion

The results obtained in the present work suggest that pharmacological modulation of β_1_AR and A_1_R activity in cardiac cells could be a potential new strategy to reduce the incidence of severe arrhythmias and increase life expectancy in PD patients.

## Declaration of Competing Interest

The authors declare no conflicts of interest.
